# Combining super-resolution microscopy with neuronal network recording using magnesium fluoride thin films as cover layer for multi-electrode array technology

**DOI:** 10.1038/s41598-019-52397-x

**Published:** 2019-11-06

**Authors:** L. Schmidl, G. Schmidl, A. Gawlik, J. Dellith, U. Hübner, V. Tympel, F. Schmidl, J. Plentz, C. Geis, H. Haselmann

**Affiliations:** 1 University Hospital Jena, Hans-Berger Department of Neurology, Section Translational Neuroimmunology, Am Klinikum 1, 07747 Jena, Germany; 2 Leibniz Institute of Photonic Technology (IPHT), Albert-Einstein-Straße 9, 07745 Jena, Germany; 3 Helmholtz Institute Jena, Froebelstieg 3, 07743 Jena, Germany; 4 Friedrich Schiller University Jena, Institute of solid state physics, Helmholtzweg 5, 07743 Jena, Germany; 5 Center for Sepsis Control and Care (CSCC), University Hospital Jena, Am Klinikum 1, 07747 Jena, Germany

**Keywords:** Biological techniques, Electrophysiology, Materials science, Materials for devices, Materials for optics

## Abstract

We present an approach for fabrication of reproducible, chemically and mechanically robust functionalized layers based on MgF_2_ thin films on thin glass substrates. These show great advantages for use in super-resolution microscopy as well as for multi-electrode-array fabrication and are especially suited for combination of these techniques. The transparency of the coated substrates with the low refractive index material is adjustable by the layer thickness and can be increased above 92%. Due to the hydrophobic and lipophilic properties of the thin crystalline MgF_2_ layers, the temporal stable adhesion needed for fixation of thin tissue, e.g. cryogenic brain slices is given. This has been tested using localization-based super-resolution microscopy with currently highest spatial resolution in light microscopy. We demonstrated that direct stochastic optical reconstruction microscopy revealed in reliable imaging of structures of central synapses by use of double immunostaining of post- (homer1 and GluA2) and presynaptic (bassoon) marker structure in a 10 µm brain slice without additional fixing of the slices. Due to the proven additional electrical insulating effect of MgF_2_ layers, surfaces of multi-electrode-arrays were coated with this material and tested by voltage-current-measurements. MgF_2_ coated multi-electrode-arrays can be used as a functionalized microscope cover slip for combination with live-cell super-resolution microscopy.

## Introduction

Super-resolution microscopy (SRM) has become increasingly important in life science in recent years. SRM overcomes the optical diffraction limit of spatial resolution in optical microscopes which is especially important for imaging of small biological substrates, e.g. localization and distributions of molecules within synapses of neurons with a diameter in a range of several hundred nanometers. The most diverse approaches or algorithms used in the techniques of stimulated emission depletion microscopy (STED)^1–3^, of direct stochastic optical reconstruction microscopy (*d*STORM)^4–7^ or of structured illumination microscopy (SIM)^8,9^ require adapted surfaces and functionalization. Beside mercaptopropyl trimethoxy silane (MPTMS), 3-Aminopropyltriethoxysilan (APTES) or other chemical routes, a standard method for functionalization of coverslip surfaces for adhesion of cells or brain slices is silanization, which is intended to realize the highest possible binding of the cells to the surface. Since the silanization procedure^10^ deals with toxic substances, safety measures are required. This functionalization must be repeated for each new experiment. For the super-resolution microscopy, especially for the *d*STORM technique used here, which represents a fluorescence microscopy method based on single molecule localization, extremely smooth surfaces with high transparency are critical. In combination with transparency measurements additionally thin coverslips are essential in order to achieve this high resolution. By the use of chemical or physical thin film deposition techniques it is possible to fabricate reproducible layers with specific properties also in mass production. Magnesium fluoride (MgF_2_) investigated in this publication is known as a standard material for coatings in optics, since it has interesting physical and chemical properties such as high hardness, good stability in hostile environments, and a very low refractive index (n = 1.38)^11–15^. It is therefore favorably used for optical components for high power lasers especially in the UV range. Due to the specific antireflective property in the visible (VIS) and near-infrared (NIR) spectral range and the very smooth layer surfaces (root mean square (RMS) = 1–5 nm), this material is suitable for use in SRM. Since the electrical isolating property of MgF_2_ is excellent it can be used as an insulating interface, for instance for multi-electrode-array (MEA) applications^16–22^. In such MEA applications the electrical activity of many cells in a network can be measured simultaneously. Therefore, use of MgF_2_ surface material combined with TCO (transparent conductive oxide) tracks and electrodes on MEAs is an innovative way to combine super-resolution imaging with functional analysis, e.g. electrophysiology^23,24^.

Here, we show the properties of the robust, functionalized surfaces based on MgF_2_ thin films prepared by electron beam evaporation (EBE) as a method for producing an electrically isolating and optically antireflective adhesion layer on very thin Borofloat glass substrates for use in biological microscopic and physiological investigations. The study includes investigation of MgF_2_ film properties and evaluates the adhesion of brain slices during immunostaining without additional fixing, since strict adherence of the tissue is mandatory for super-resolution imaging. Therefore, a comparison between silanized and MgF_2_-covered substrates using epi-fluorescence and *d*STORM is presented. Moreover, a technology flowchart for ITO/MgF_2_-MEAs was developed and resulting properties are tested.

## Materials and Methods

### Thin film preparation and electrode structuring

Using an EBE system, the MgF_2_ layers were deposited on Borofloat glass substrates (Schott AG, thickness 700 µm) for investigating the influence of substrate temperature and layer thickness on film microstructure, transmittance and film crystallinity. The thickness was varied from 110 nm to 350 nm and could be monitored by a quartz oscillator during the deposition. Beside the film thickness, the substrate temperature was also varied from ambient temperature (AT) to 400 °C, in order to change the structural properties of the films. The transformation temperature of the glass substrates, which lies at 557 °C, must be noted. The glass substrates (R.Langenbrinck GmbH) used in epi-fluorescence and super-resolution microscopy experiments had a diameter of 18 mm and a thickness of 170 µm. These thin substrates should avoid aberrations. All substrates were cleaned with acetone, isopropanol and ultra-sonic before deposition.

The MEAs consist of two film types. The ITO films (indium tin oxid, 200 nm film thickness), the electrical track material, were deposited by DC sputtering using an ITO 90:10% target. The structuring of the ITO electrodes and the tracks was performed by photolithography and ion beam etching. After the following deposition of the isolating MgF_2_ layer the ITO electrodes were uncovered by a photolithography and a subsequent dry etching process. The diameter of the ITO electrodes is 30 µm in size and they have a distance of 200 µm. Here, 700 µm thick Borofloat glass substrates were used to evaluate the structuring procedure in a first step.

### Thin film characterization methods

For morphological analysis including quantitative analysis of the film surface roughness, we employed atomic force microscopy (AFM, *Dimension Edge, BRUKER*). For observation of cross-sectional images we used field-emission scanning electron microscopy (*FEI Helios NanoLab G3 UC, ThermoFisher Scientific*). The AFM is operated in tapping mode with a tapping tip (*Tap300Al-G; Bidget Sensors*) covered with an Al-reflection layer (tip radius below 10 nm). The bigger electrode structures can be inspected by light microscopy.

The crystalline orientation of the MgF_2_ layers was investigated by X-ray diffraction (XRD, *Panalytical X’Pert Pro* for crystallite size estimation) with Cu-Kα_1,2_ (Kα_1_: 1.5406 Å) radiation. In order to characterize the conductivity properties of the layers and the electrode structures, 4-point measurements with gold pins were carried out. A computer-supported measuring system (*Labview, National Instruments*) with a source-measurement-unit (*SMU238, Keithley*) was being used for data read out.

### Immunostaining of 10 µm brain slices

Native mouse brain tissue was harvested and snap frozen in TissueTek embedding compound (*Sakura*) at −80 °C. 10 µm coronar cryosections were prepared with a cryotome (*CM3050S, Leica, Wetzlar, Germany*).

The slices were transferred to cover slips (silanized or MgF_2_ covered glass substrates) for optical detection by immunostaining. For immunostaining the slices were dried for 1 h, incubated for 5 min with DAPI (4′,6-Diamidin-2-phenylindol) solution and washed three times for 5 min in PBS (Phosphate-buffered saline).

Afterwards, brain slices were blocked for 2 h with the blocking solution (*BS*, 10% bovine serum albumin/10% normal donkey serum/10% normal goat serum/PBS, 0.1% Triton X-100) at room temperature (RT) followed by an incubation with the first primary antibody (rabbit anti-Homer1 from *Synaptic Systems*, 1:400, *#160003* or mouse anti-GluA2 from *Millipore*, 1:100, *MAB397*) in the incubation solution (*IS*, 1% bovine serum albumin/1% normal donkey serum/1% normal goat serum/PBS, 0.1% Triton X-100 and for GluA2 without 0.1% Triton X-100) overnight at 4 °C.

After washing steps at RT the first secondary antibody (anti-rabbit AlexaFluor 647 from *Life Technologies*, 1:200, *#A21246* or anti-mouse AlexaFluor 647 from *Life Technologies*, 1:200, *#A21235*) was incubated followed by washing steps and blocking with PBS (phosphate buffered saline) for 2 h at RT.

The second primary antibody (guinea pig anti-Bassoon from *Synaptic Systems*, 1:400, *#141004* or rabbit anti-Homer1 from *Synaptic Systems*, 1:400, *#160003*) was incubated in *IS* with 0.1% Triton X-100 overnight at 4 °C. On the next day the slices were washed and incubated with the secondary antibody (anti-guinea pig CF568 from *Biotium*, 1:200, *#20377–500* *uL* or anti-rabbit CF568 from *Biotium*, 1:200, *#20098*) for 2 h at RT followed by additional washing steps.

Tissue harvesting was approved by the Thuringian state authorities (TWZ-28-2017). We have followed the animal care guidelines of the University Hospital of Jena during our study.

### Optical recording and *d*STORM imaging

The optical characterization of the prepared films with respect to transmission, reflectivity and absorption was carried out by a spectrometer with an integrating sphere (*Lambda 900, Perkin Elmer Instruments*).

The application of the MgF_2_ films was evaluated by overview brain slice images recorded in phosphate-buffered saline (PBS) by epi-fluorescence. All recording parameters (pixel size, exposure time, gain, light power) were kept constant during the experiments. The filter sets (DAPI signal FilterSet 49 and AlexaFluor 647 FilterSet 50 from Zeiss) were used for epi-fluorescence images. Afterwards, the same brain slices were recorded with *d*STORM a localization-based super-resolution fluorescence microscopy (*Elyra P.1, Zeiss Microscopy*) with a localization precision of up to 10 nm. *d*STORM experiments were performed in MEA buffer containing 100 mM mercaptoethyl-amine (ph adjusted to 7.9) and by the laser lines of 561 nm and 642 nm (Lasos Lasertechnik, Jena, Germany). All imaging settings were kept constant over all experiments. Fluorophores were sequentially recorded starting with longer wavelength and using a 1.46 NA 100x TIRF oil objective (Zeiss, Jena Germany). 100% of laser power of both laser lines was constantly applied during the experiments. For each channel 25000 frames were recorded with a frame size of 160 × 160 pixels applying a camera exposure time of 15 ms and in-software detector gain of 150. The sample z-drift was stabilized using Definite Focus (Zeiss, Jena, Germany). Residual drift in x-y direction was corrected using a model based on drift algorithm implemented in *ZEN 2009 software* (*Zeiss, Jena, Germany*). Chromatic aberration was corrected by performing a channel alignment using tetraspeck beads.

### Ethical approval

Tissue harvesting for the optic experiments was approved by the Thuringian state authorities (TWZ-28-2017). We have followed the animal care guidelines of the University Hospital of Jena during our study.

## Results and Discussion

### Structural properties of the MgF_2_ thin films

The structural properties, such as crystallinity, grain size, and crystal orientation of a layer, are strongly related to the growth conditions, and thus to the deposition parameters. These growth conditions influence the surface properties, and therefore the aging process as well as the features of the layers under ambient conditions. This is important for biological applications because the layers have to resist repetitive washing procedures. Furthermore, they need to be also chemically resistant for reutilization. Very dense layers are necessary to prevent the penetration of water and thus layer degradation. Smooth or rough layers could be responsible for good or bad adhesion of slices or growing of cell cultures, respectively. For this reason, the layer production by means of high-energy physical and chemical deposition methods, e.g. electron beam evaporation, is advantageous. According to the structure zone model (SZM) of Movchan and Demchishin^25^ for evaporation the layer microstructure is influenced by the deposition temperature, i.e. by the ratio of substrate temperature (Ts) and melting temperature (Tm) of the material. The growth model scheme is divided into 3 structure zones (zone 1: Ts < 0.3 Tm, zone 2: 0.3 Tm < Ts < 0.5 Tm; zone 3: 0.5 Tm < Ts, Tm (MgF_2_) = 1536 K).

**Figure 1:**
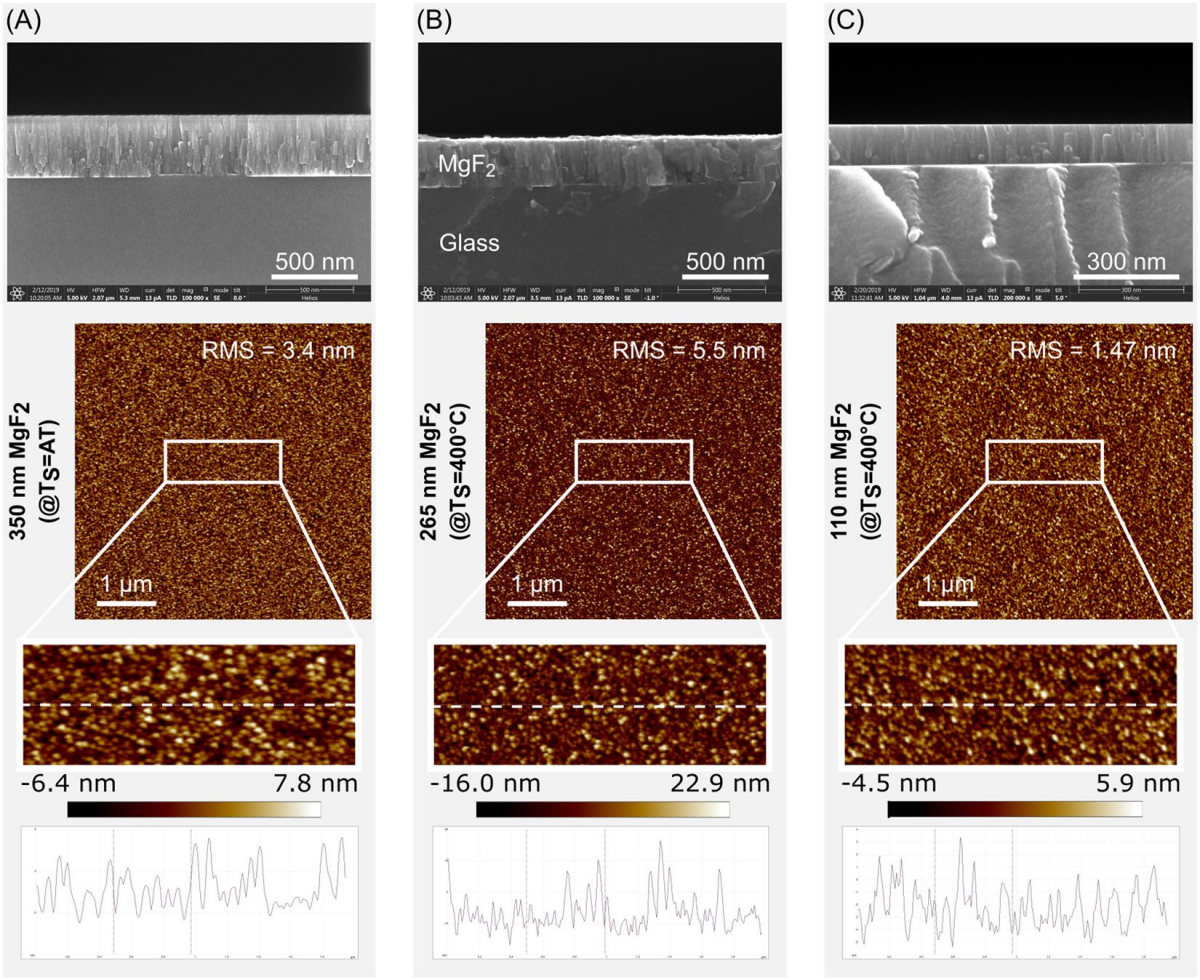
Top row: Cross-sectional SEM images of MgF_2_ films using an acceleration voltage of 5 kV. Bottom row: AFM images (5 × 5 µm^2^) with RMS roughness values of the film surfaces and zoomed images with the corresponding height profiles. The brightness illustrates the surface morphology of the MgF_2_ films. The MgF_2_ films were fabricated with different thickness on different heated glass substrates. **(A)** d = 350 nm, Ts = ambient temperature (AT), (**B**) d = 265 nm, Ts = 400 °C, **(C)** d = 110 nm, Ts = 400 °C.

AFM as well as cross-sectional SEM images show the values of roughness and film structure development dependent on substrate temperature and film thickness (Fig. 1). Since we performed the deposition of the layers at substrate temperatures equal or below 400 °C, the resulting layer structure can be described by structure zone 1 or zone 2. The films of zone 1 (Fig. 1(A), Ts = AT, Ts/Tm = 0.19) are characterized by a column-shaped structure with fine fibre structures separated by voided boundaries. The columns are generally not single grains. They can consist of smaller grains or can even be amorphous. The growing is determined by insufficient adatom diffusion but more by atomic shadowing. Such a growth indicates a porous structure. MgF_2_ films deposited at 400 °C (Fig. 1(B,C), Ts = 400 °C, Ts/Tm = 0.44) already belong to zone 2. Broader cone shaped columnar grains also develop there, but the microstructure is less voided than in zone 1. Furthermore, it is known, that thicker layers are usually leading to rougher surfaces which can be observed comparing Fig. 1(B,C). Of note, the model can slightly vary by using different materials.

Previously it was demonstrated that water incorporation is responsible for the aging of the layers^14^. However, this phenomenon is reversible when the sample is placed in vacuum. As a result, film production at high temperatures with a small layer thickness of about 110 nm offers advantages and is best placed for biological approaches.

**Figure 2:**
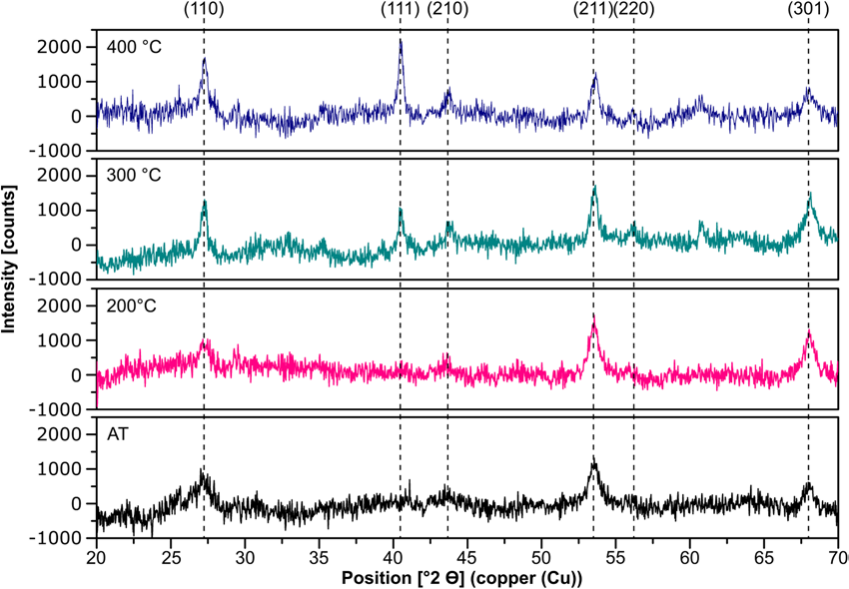
*θ*-2*θ* scan of MgF_2_ films prepared at four different substrate temperatures. The XRD-signals of the amorphous glass were subtracted. Assignment of the diffraction reflexes to the lattice planes (hkl).

Additionally, the crystallinity of the films should be considered which is also influenced by the deposition conditions^14,15^. The x-ray diffraction (XRD) pattern (reflex position, height and width) provides information about structural material properties (e.g. grain sizes, defects) which can influence optical as well as electrical properties and chemical behavior. To illustrate the differences in crystallinity we present four samples of XRD spectra of the MgF_2_ films corrected by the amorphous glass substrate (Fig. 2). The peak positions of the different MgF_2_ films observed at 27.3°, 40.5°, 43.7°, 53.6°, 56.2° and 68.0° correspond to the known (110), (111), (210), (211), (220) and (301) reflexes. The crystal structure of the films deposited on silica substrates at ambient temperature (AT) is polycrystalline showing weak (110), (211) and (301) peaks, whereas at higher temperatures (Ts ≥ 300 °C) the polycrystalline structure develops a pronounced (111) texture and the reflex of the (110) lattice plane increases. The reflexes become narrower with an increase in deposition temperature. That means a changing in crystallite size and defects.

**Figure 3:**
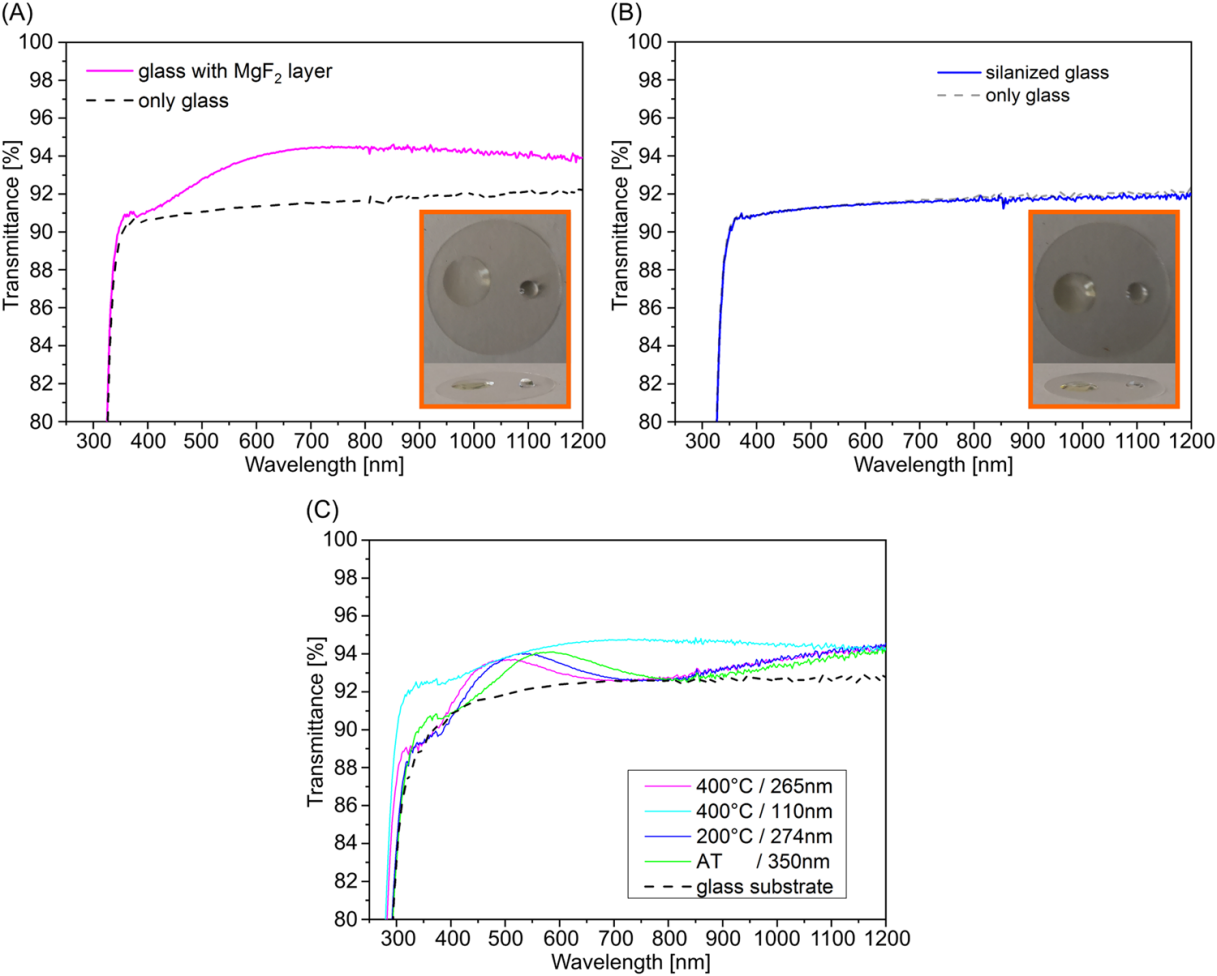
Comparison of transmission spectra of (**A**) a MgF_2_ covered glass (Ts = 400 °C, d_layer_ = 110 nm) and (**B**) a silanized glass. Photos: wetting properties, water (right) and oil (left) droplets. (**C**) Transmittance spectra of MgF_2_ films dependent on substrate temperature during deposition and on film thickness.

**Figure 4:**
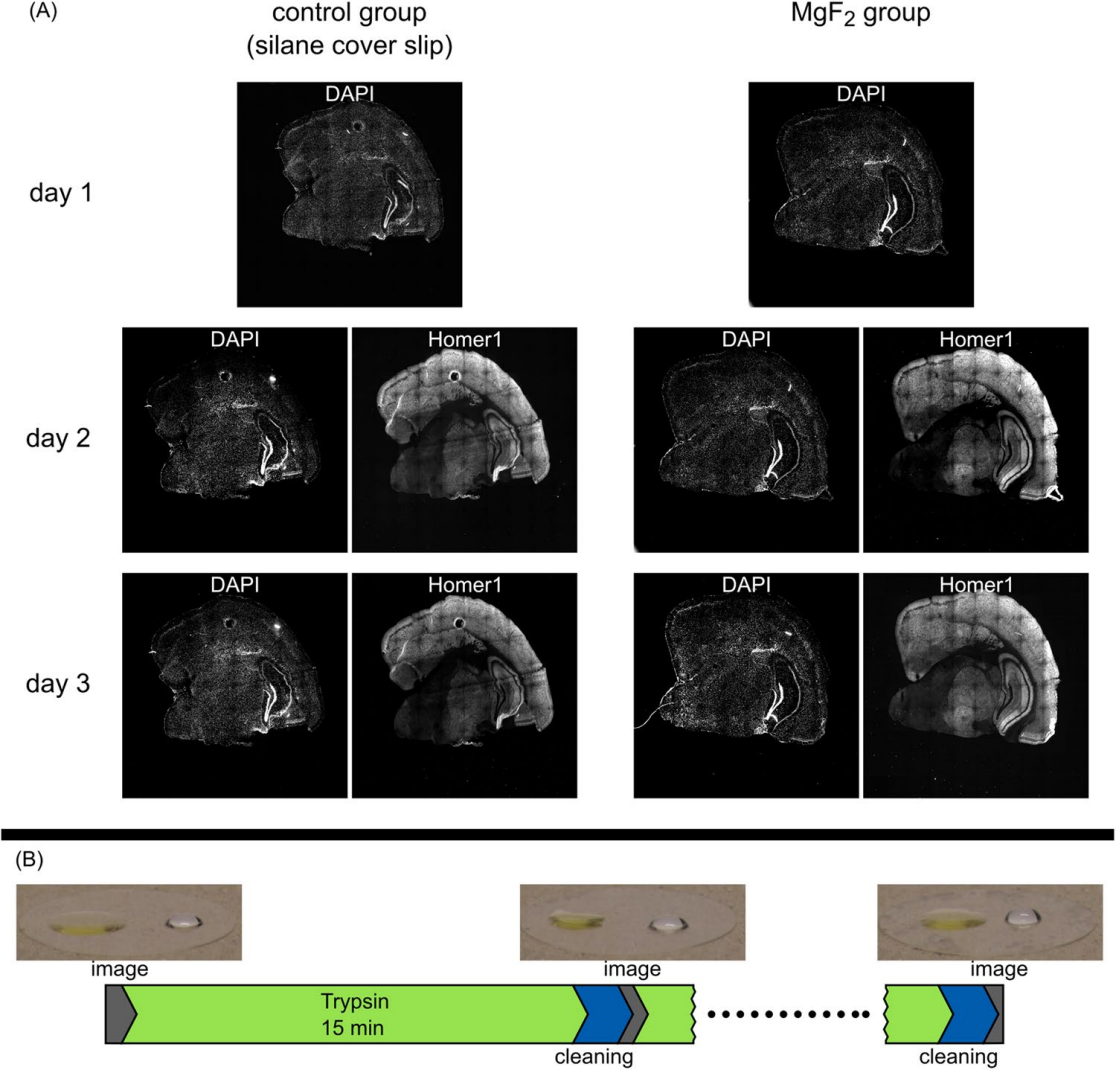
(**A**) Comparison of epi-fluorescence images of mouse brain slices after staining with DAPI (day 1 to 3) and homer1 (from day 2) on MgF_2_ coated and silanized cover glasses. Observation over 3 days. (**B**) Stable wetting properties after cleaning, water (right) and oil (left) droplets. Time sequence visualization of a washing process with trypsin and cleaning steps with acetone, methanol and water. Example pictures of droplets after the cleaning process. The last picture was taken after 10 washing cycles.

Furthermore, the MgF_2_ layer showed the desired hydrophobic and lipophilic properties analogous to silanized coverslips. This was tested by a water or oil droplet as demonstrated in Figs 3 and 4. Together, these features seem favorable for adhesion of biological material without additional fixation, e.g. for brain slices.

### Optical properties of the MgF_2_ thin films

In addition to the electrically insulating effect of MgF_2_ layers, the optical properties are especially important for the use in super-resolution microscopy. Not only very smooth layers but also layers with low losses are required in order to increase the luminous efficiency and the optical resolution. Coating with a thin MgF_2_ layer (110 nm) increases the transmission in both the VIS and the NIR spectral range compared to the uncoated substrate and to the silanized substrate (Fig. 3(A,B)). Therefore, thin MgF_2_ layers are often used as anti-reflecting layers in optics and are also suitable for use in SRM in both VIS and NIR spectral range.

Moreover, the transparency of the layer can be adjusted in a desired wavelength range by the layer thickness (Fig. 3). Thus, the thickness of the layer, i.e. the transparency range, can be selected according to the application. Highest transparency over a wide spectral range can be achieved by thin films, e.g. d = 110 nm and are optimal from our point of view. Spectral test experiments at 561 nm and 642 nm also showed no detectable autofluorescence of the MgF_2_ layers.

### *d*STORM imaging of brain slices adherent on MgF_2_ thin films

In a first step, the adhesion of brain slices to the MgF_2_ coated cover glasses (MgF_2_ group) was investigated in comparison to silanized cover glasses (control group). For these experiments 170 µm thick glass substrates with a 265 nm as well as a 110 nm thick MgF_2_ layer were used. Adhesion of brain slices was investigated using epi-fluorescence microscopy of cell nuclei (DAPI) stains on day 1 after tissue mounting and of DAPI and postsynaptic homer1 immunofluorescence after primary and secondary antibody staining on day 2 and 3. Brain slice adhesion was comparable in MgF_2_ coated and silanized cover glasses (Fig. 4). Even after staining procedures with intensive washing steps no structural changes have been detected, indicating proper adhesion of brain slices in solution over several days. Other than silanized cover slips, MgF_2_ coated cover glasses are reusable after removal of brain slices, cleaning, and vacuum treatment.

**Figure 5:**
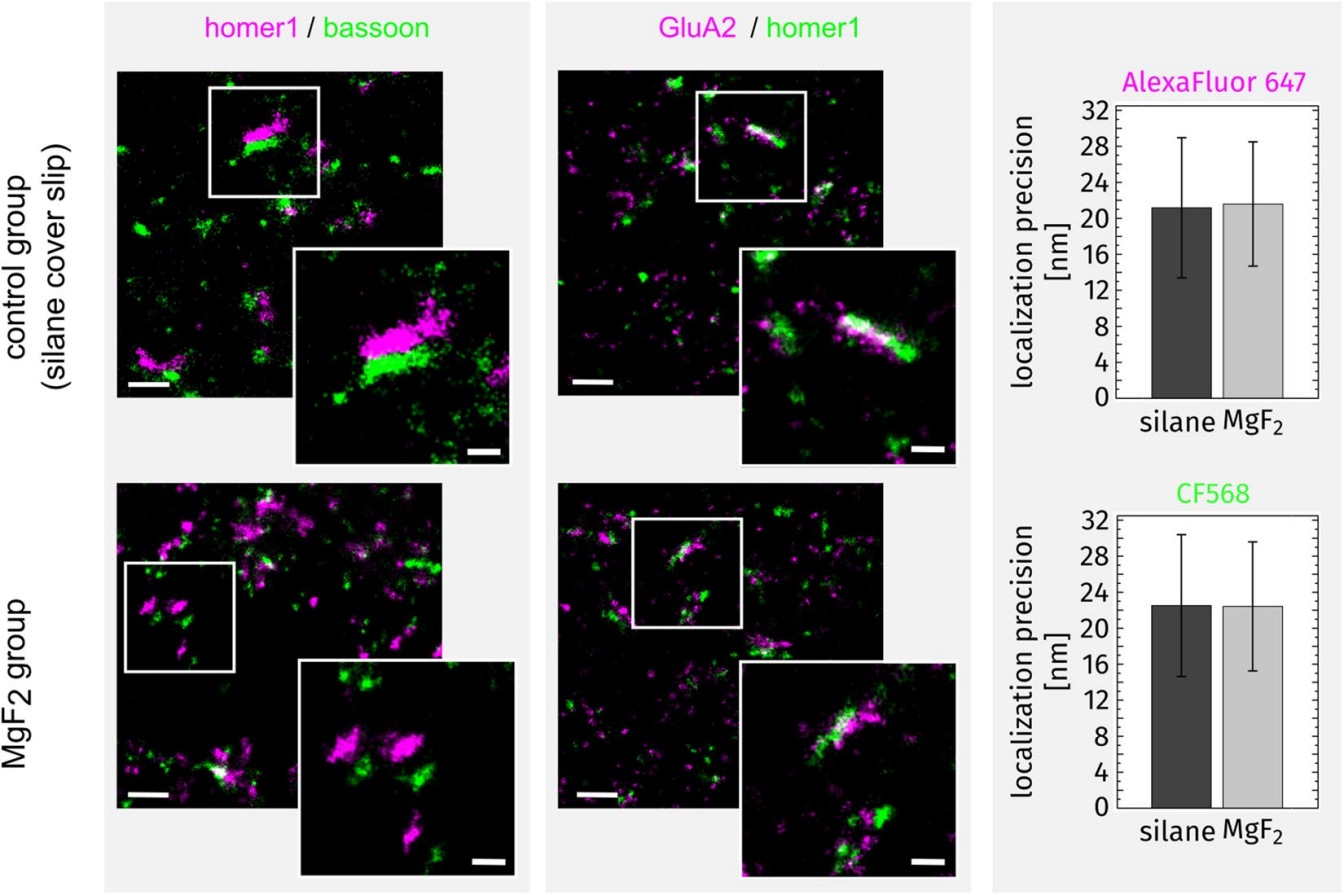
*d*STORM images of brain slices after reconstruction. MgF_2_ coated (110 nm) and silanized cover glasses (MgF_2_ group and control group, respectively). (Scale bar overview images 500 nm; scale bar insets 200 nm; magenta shows the postsynaptic marker homer1 (left column) and the AMPA receptor subunit GluA2 (middle column), green shows the presynaptic marker bassoon (left column) and postsynaptic marker homer1 (middle column)). Right column shows the localization precision of the two dyes AlexaFluor 647 (magenta) and CF568 (green).

In the second step we evaluated such layered cover glasses for use in super-resolution microscopy. We applied single-molecule localization based *d*STORM because this provides highest possible lateral resolution in light microscopy up to 20 nm. We investigated brain tissue stains with the postsynaptic marker homer1 and the presynaptic marker bassoon to evaluate if super-resolution imaging with the MgF_2_ coated and silanized cover glasses is able to resolve small pre- and postsynaptic structures in brain slices. Furthermore, by co-staining of homer1 and the AMPA receptor GluA2 subunit we tested receptor co-localization in the postsynaptic field as well the resolution of small structures. We found identical results in both experimental groups with well-resolved synaptic structures indicating similar properties for super-resolution imaging in MgF_2_ coated in comparison to conventional silanized cover glasses (Fig. 5).

### MgF_2_ layers as electrical isolating top layer for multi-electrode-arrays

**Figure 6:**
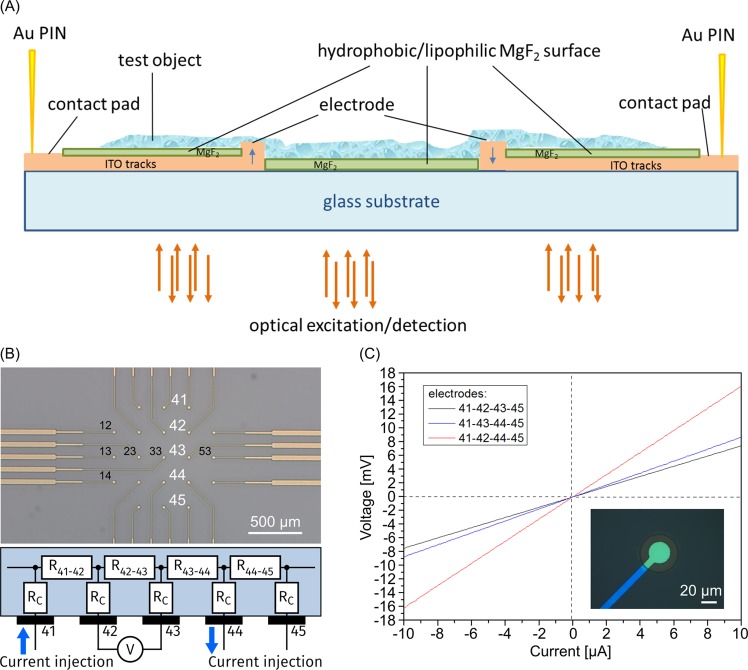
(**A**) Schematic cross-sectional drawing of an MEA-Chip; (**B**) Overview of the used MEA-Test-Chip with tested electrodes (41, 42, 43, 44, 45); Electrode and track material: ITO; Isolation and function layer material: MgF_2_; Substrate: 25 × 25 mm glass chip, 700 µm thickness; circuit diagram for the four-point measurements, R_C_ = contact resistance, R_4×4y_ = measuring resistance; (**C**) V-I-characteristic curves for one line (see (B)); first and last electrodes for current injection, middle electrodes for voltage measurement; inset: ITO electrode 30 µm diameter (green) and MgF_2_-covered track (blue).

Next, we sought to develop special MEAs for electrophysiological investigations of cell activity in neurological networks using thin MgF_2_ coatings. These might be useful for several applications in biomedical research, e.g. to investigate neuronal networks and synaptic function and pathophysiological changes thereof in dissociated primary neurons and in brain sections. The novel, highly transparent, and functionalized design of the MEA structures enables the simultaneous use of microscopy and electrophysiology. For this combination the MEA chip requires a thin substrate (glass cover slip in the range of 170 µm) and a very thin isolating coating on the tracks with a very smooth surface in order to guarantee high transparency and spatial resolution without aberrations. In order to establish the film structuring and to verify the electrical functionality we used thicker substrates (glass cover slips of 700 µm thickness) in a first step. A schematic cross-sectional drawing of an MEA-Chip structure is shown in Fig. 6(A).

We prepared a standard square electrode array structure for testing the flow chart of chip structure preparation (Fig. 6(B)). The electrodes are 30 µm in size and have a distance of 200 µm. The electrode arrangement and size can be adapted to the favored application and should be as close as possible to each other to improve spatial resolution. The tracks were covered by the electrically isolating MgF_2_ film and the electrodes were exposed by etching (Fig. 6(C), inset image). The isolation effect of MgF_2_ films was tested by film resistivity measurements.

The functionality of the array electrodes was tested in a next step by measurements using the 4-point geometry. A conductive foil was used as a test object, which was placed on the surface of the array for simulation of bath solution. This foil has the advantage of having a relatively homogeneous resistivity. Using for example the electrodes (41, 42, 43, 44, 45; Fig. 6(B)), which are lined up in a row, the linearity of the V-I curve can be observed (Fig. 6(C)), indicating ohmic behavior. For these measurements a chip-adapted-contact-pad, consisting of a circuit board and gold pins was developed, which allows the 4-point measurements for all electrode configurations. A relatively low-noise measurement was possible in this 4-point geometry despite the very high contact resistance of the test foil of 80–100 kΩ between two pads. When current was applied to electrodes 41 and 45 the resistance R_42–44_ was 1599 Ω between the electrodes 42–44 (Fig. 6(C) red line) and emerged from the addition of the resistances R_42–43_ = 700 Ω and R_43–44_ = 858 Ω, as expected with a series connection (Fig. 6(C) black and blue line, respectively). In this way, we have tested the functionality of all contact pads on the chip. Moreover, we could not detect any leakage currents in test measurements with aqueous puffer for film thickness above 300 nm. Thus, the functional feasibility has been demonstrated. Future experiments will be carried out using thinner MgF_2_ layers and thinner autofluorescence-free glass substrates for the investigation of biological samples finally resulting in a combination of optical and electrophysiological methods.

## Conclusions

We found that a small layer thickness of about 110 nm on ultra-thin cover glasses and a roughness of less than 5 nm prevent aberrations and are therefore excellently suited for the use in SRM. We demonstrated that the deposition temperature of MgF_2_ is responsible for a dense growing of the films. The surface roughness is especially dependent on the film thickness and lies between 1 and 5 nm for the investigated layers.

In contrast to silanized cover glasses, the robust and already functionalized surfaces of cover glasses with MgF_2_ layer are reusable and can be reproducible fabricated without toxic starting reagents. In addition, they also show hydrophobic and lipophilic properties with very good adhesion of brain sections without additional fixing over days after staining and washing processes.

The transparency in a certain wavelength range from 300 nm to 6 µm can be adjusted via the layer thickness. MgF_2_ layers simultaneously serve as anti-reflective layers and therefore show a transparency higher than the glass substrate, which is advantageous for the light yield in microscopy. Due to the deposition at higher temperatures, the absorption edge given by the glass is also slightly displaced, which points to an influence on the used substrate. Of course, the softening temperature of glass must be taken into account.

We demonstrated these methodical advantages using localization-based super-resolution *d*STORM with very high lateral resolution in 10 µm brain slices without additional fixing of the slices on MgF_2_ coated cover glasses. Imaging of central synapses by use of two-color *d*STORM of a post- and presynaptic marker structure resulted in excellent imaging of these closely located biological structures indistinguishable from conventional silanization procedures.

These characteristics of MgF_2_ layers in combination with TCO tracks offer the unique opportunity for fabrication of MEAs that are especially suited for simultaneous use of high resolution microscopy together with electrophysiological recordings. On basis of the coating and material studies we are able to fabricate appropriate MEAs. The aspired objective is to structure the MEAs on autofluorescence-free glass substrates of 170 µm thickness and thinner to avoid aberrations in microscopy.

The combination of super-resolution microscopy and MEA-network analysis is particularly useful for live cell analysis. The combined investigation method can be used for the analysis of cell cultures and brain slices using several super-resolution methods including *d*STORM, PALM, SIM, Lattice SIM or STED. SIM and MEAs can be applied for investigation of cell cultures or thin slices. Lattice SIM or STED together with MEAs would be interesting for analysis of cell cultures and brain slices up to a thickness of 200 µm. It would also be conceivable to embed nanoparticles in such high optically transparent layer of MgF_2_ for drift corrections in super-resolution microscopy applications for long-term experiments.

In summary, this approach may serve as a basis for further developments with respect to the simultaneous use of optical and electrophysiological *in-vitro* experiments. The possibility to correlate microscopy with electrophysiology is an important step towards understanding the molecular mechanisms of neuronal and synaptic function and of molecular pathophysiology in neurological diseases.
